# Supplementary material for: The Effectiveness of a Novel Air-Barrier Device for Aerosol Reduction in a Dental Environment: Computational Fluid Dynamics Simulation
Source: Bioengineering (Basel). 2023 Aug 8;10(8):947. doi: 10.3390/bioengineering10080947 (PMC10452020; doi:10.3390/bioengineering10080947)
Supplement: Supplementary file 1 [file bioengineering-10-00947-s001.zip › bioengineering-2484561-supplementary.pdf]

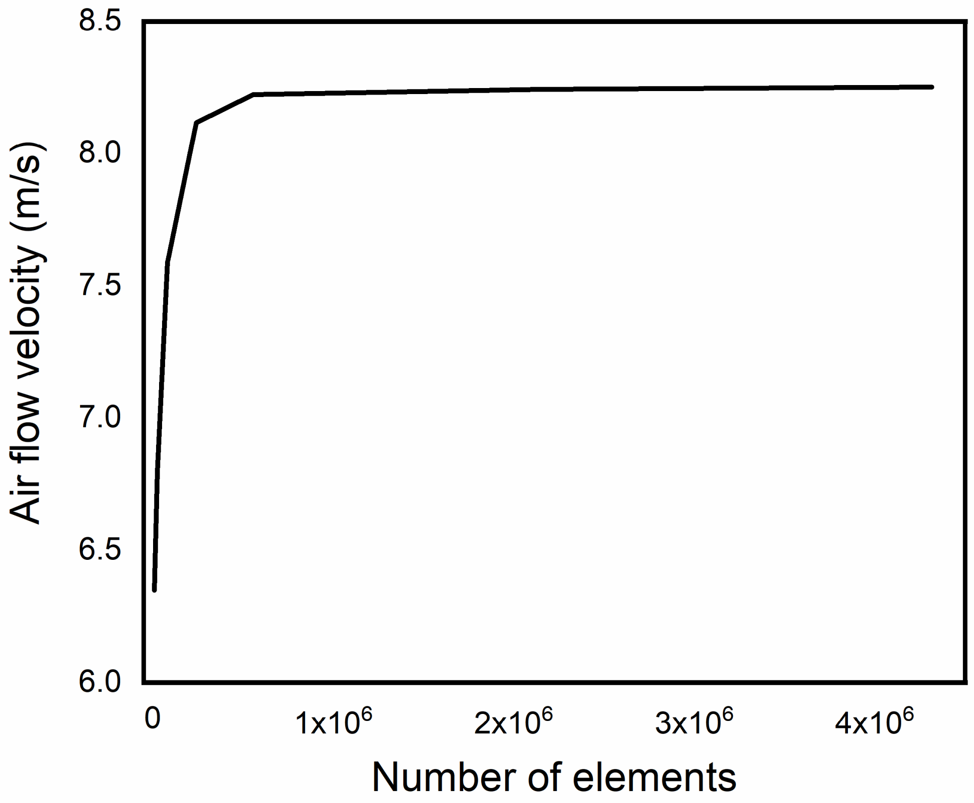

Figure S1. The convergence and mesh independence of study of COMSOL simulation.

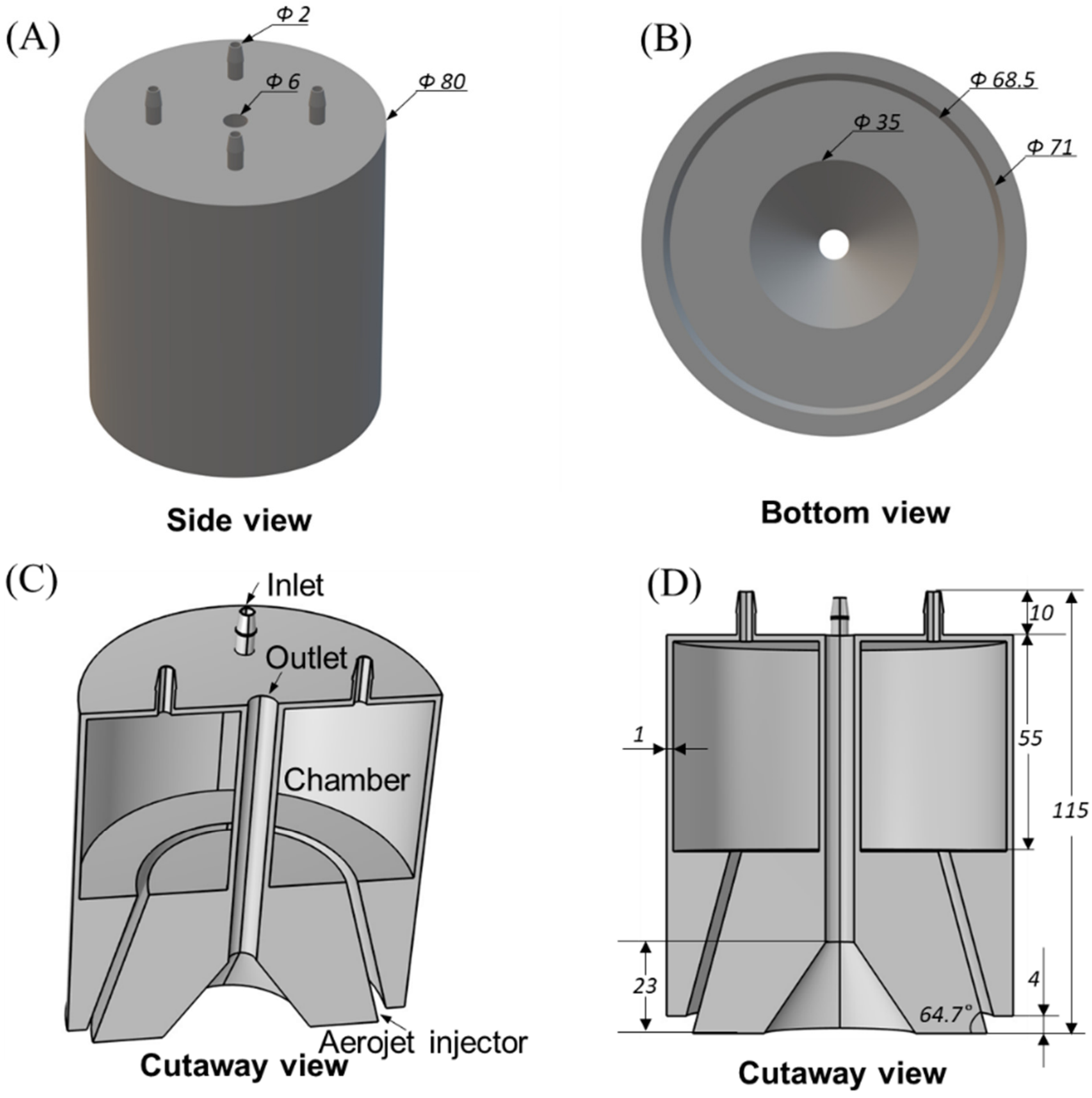

Figure S2. The detailed design and dimensions of the proposed device in COMSOL simulation.

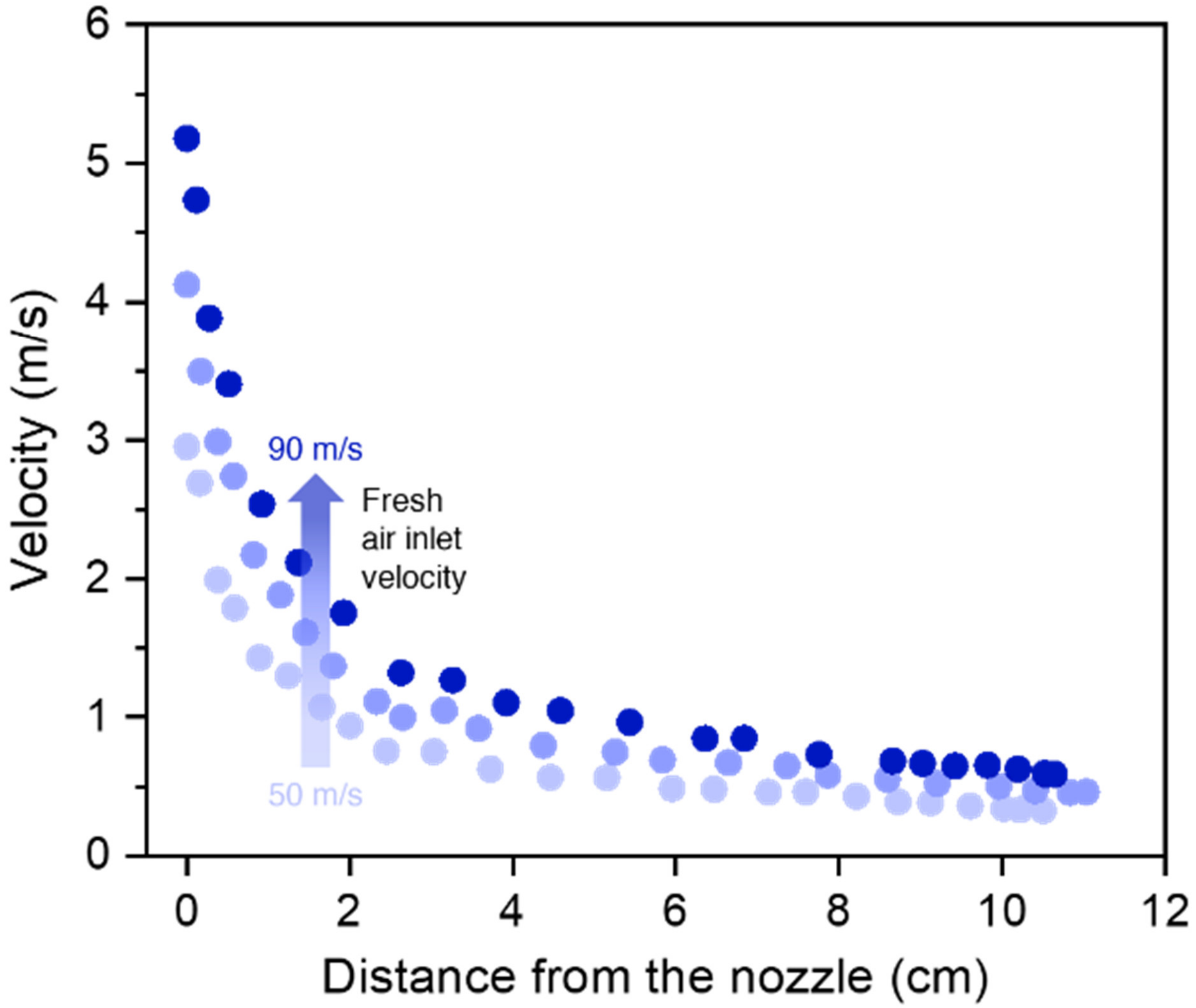

Figure S3. Velocity profiles of the air barrier at varied fresh-air inlet velocities (no ventilation applied).

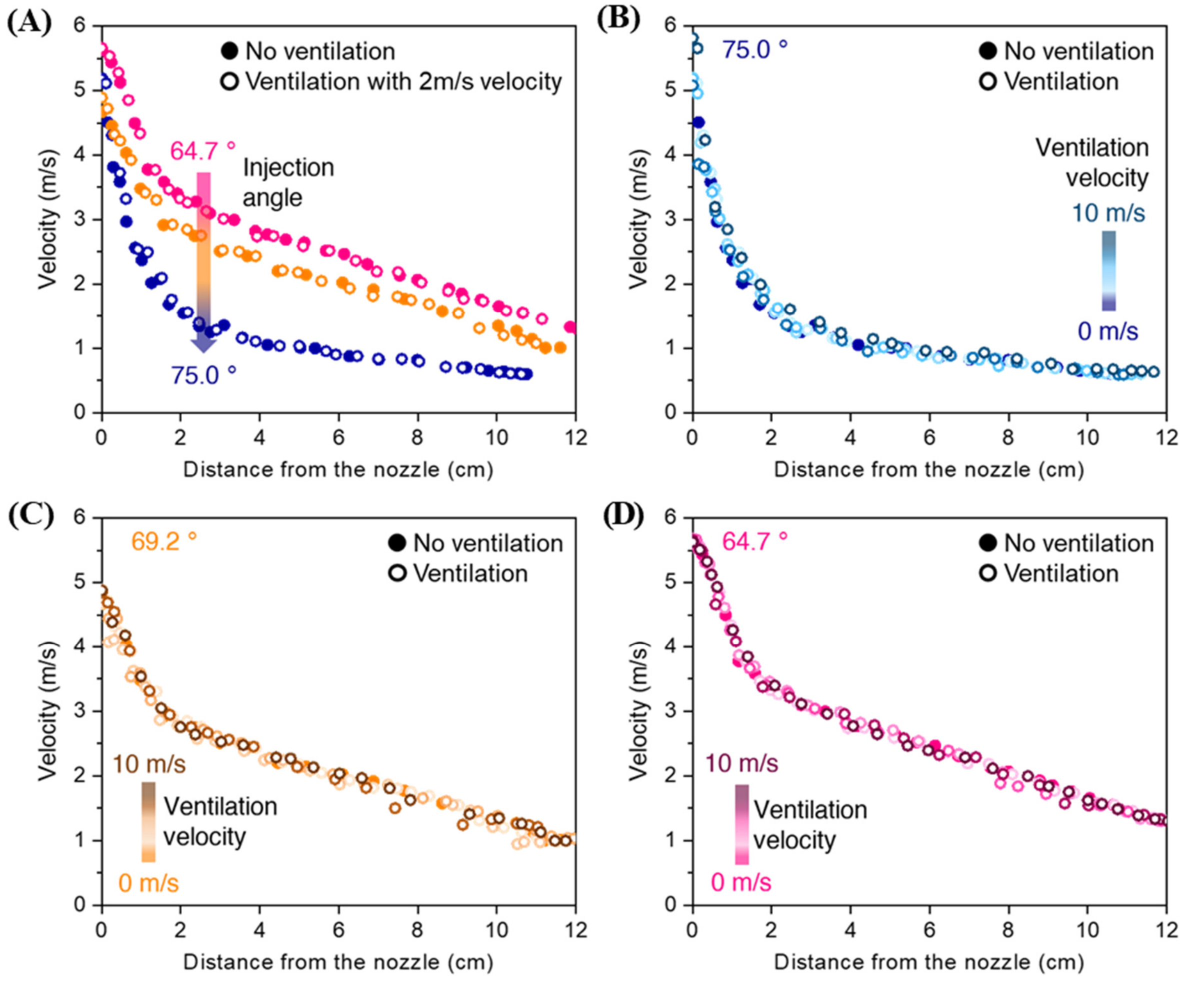

Figure S4. (A) The velocity of the air barrier as a function of distance from the nozzle with and without ventilation of 2 m/s. Velocity profiles of the air barrier with different ventilation velocities (0, 2, 4, 6, 8, 10 m/s) at **(B)** 75°, **(C)** 69.2°, and **(D)** 64.7°.

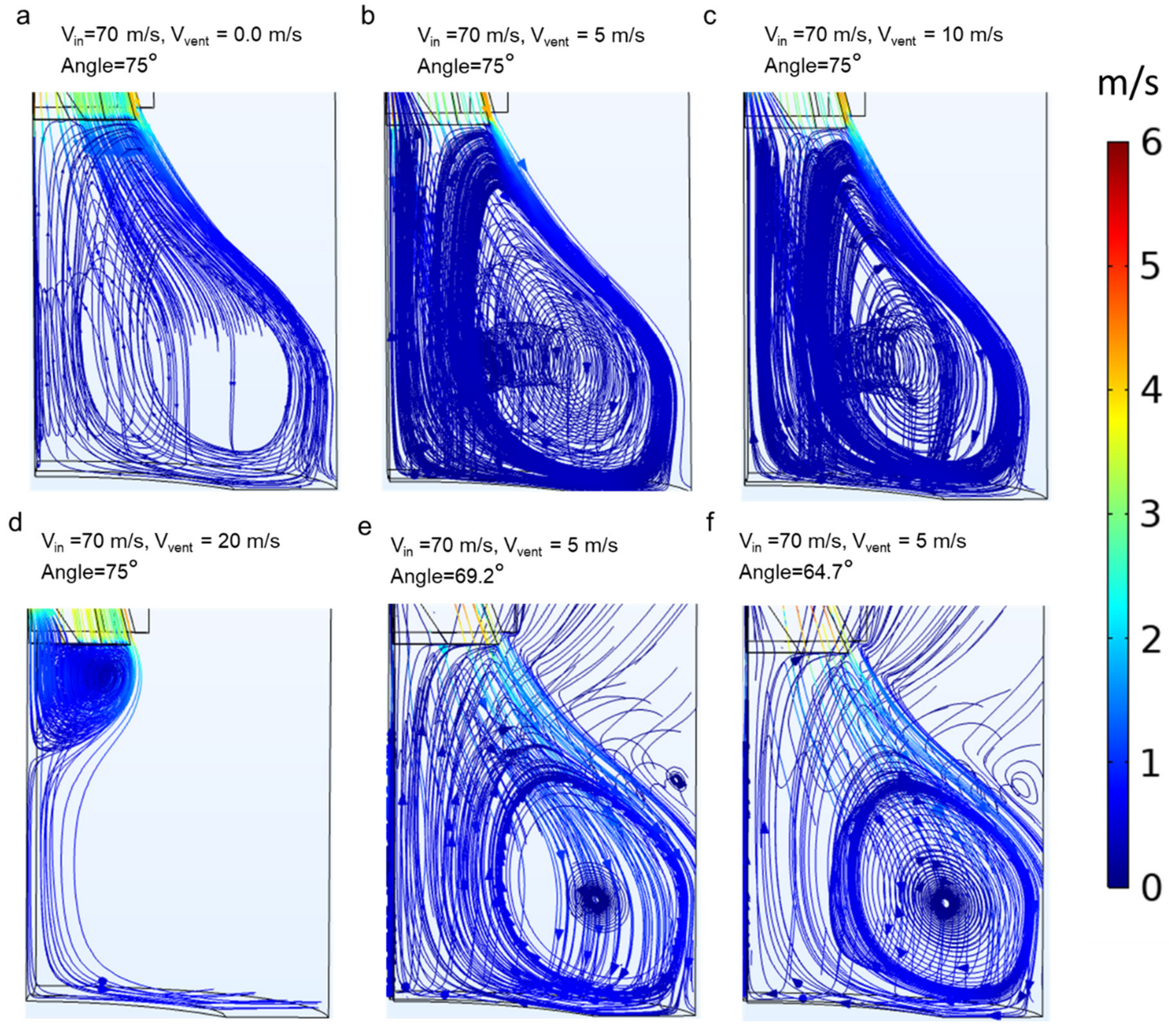

Figure S5. The streamline in the patient zone in the presence of the air barrier with varied ventilation velocities of **(A)** 0 m/s, **(B)** 5 m/s, **(C)** 10 m/s, and **(D)** 20 m/s and different injection angles of (**E**) 69.2°, (**F**) 64.7°.

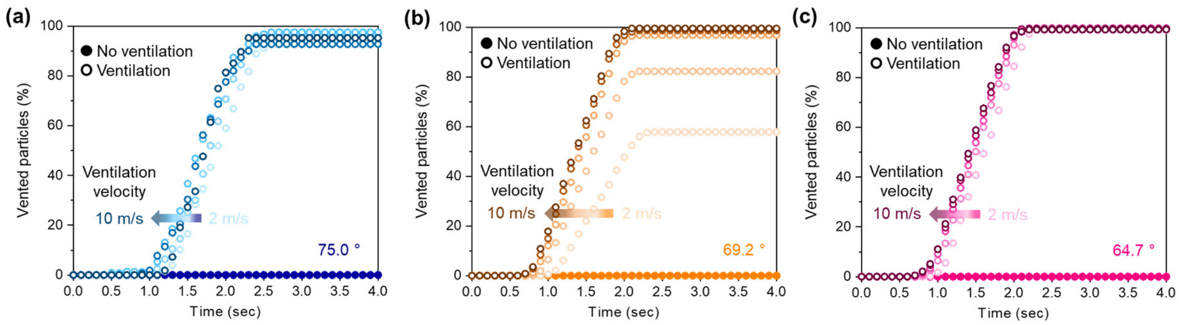

Figure S6. Vented particles (%) with different ventilation velocities (0, 2, 4, 6, 8, 10 m/s) at **(A)** 75°, **(B)** 69.2°, and **(C)** 64.7°.

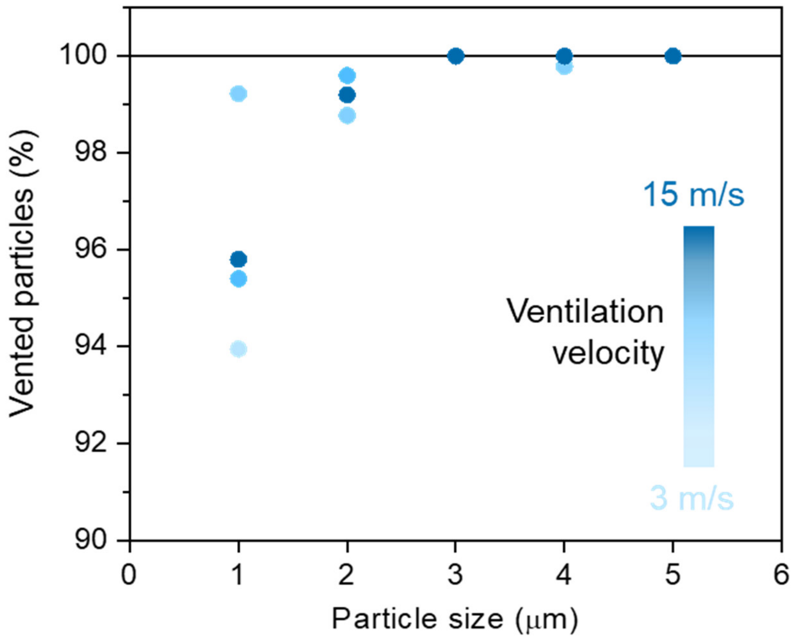

Figure S7. The vented particles (%) of microparticles in the range of 1 μm to 5 μm at different ventilation velocities (3, 7, 11, 15 m/s).

Table S1. The contaminant removal effectiveness at varied injection angles, injection velocities, and ventilation velocities.

Injection angle (°)	Injection velocity (m s^{-1})	Ventilation velocity (m s^{-1})	CRE
75.0	3.1	5	464
75.0	3.6		1569
75.0	4.1		766
75.0	5.1		4819
64.7	5.1	6	1310
69.2			1000
75.0			16300
